# “Waitlist mortality” is high for myeloma patients with limited access to BCMA therapy

**DOI:** 10.3389/fonc.2023.1206715

**Published:** 2023-08-03

**Authors:** Nausheen Ahmed, William Wesson, Muhammad Umair Mushtaq, Rajat Bansal, Haitham AbdelHakim, Sarah Bromert, Allison Appenfeller, Batool Abu Ghazal, Anurag Singh, Sunil Abhyankar, Siddhartha Ganguly, Joseph McGuirk, Al-Ola Abdallah, Leyla Shune

**Affiliations:** ^1^ Division of Hematologic Malignancies & Cellular Therapeutics, University of Kansas Cancer Center, Westwood, KS, United States; ^2^ School of Medicine, University of Kansas, Kansas, KS, United States; ^3^ Mary and Ron Neal Cancer Center, Houston Methodist Hospital, Houston, TX, United States

**Keywords:** BCMA, ide-cel, access, production slot, waitlist, myeloma

## Abstract

**Background:**

The first-in-class approved BCMA CAR-T therapy was idecabtagene vicleucel (ide-cel), approved in March 2021, for RRMM patients who progressed after 4 or more lines of therapy. Despite the promising outcomes, there were limited apheresis/production slots for ide-cel. We report outcomes of patients at our institution who were on the “waitlist” to receive ide-cel in 2021 and who could not secure a slot.

**Methods:**

We conducted a retrospective review of RRMM patients evaluated at the University of Kansas Cancer Center for ide-cel from 3/2021-7/2021. A retrospective chart review was performed to determine patient and disease characteristics. Descriptive statistics were reported using medians for continuous variables. Survival analysis from initial consult was performed using Kaplan-Meier Survival estimator.

**Results:**

Forty patients were eligible and were on the “waitlist” for CAR-T. The median follow-up was 14 months (2-25mo). Twenty-four patients (60%) secured a production slot and 16 (40%) did not. The median time from consult to collection was 38 days (8-703). The median time from collection to infusion was 42 days (34-132 days). The median overall survival was higher in the CAR-T group (NR vs 9 mo, p<0.001).

**Conclusion(s):**

Many patients who were eligible for ide-cel were not able to secure a timely slot in 2021. Mortality was higher in this group, due to a lack of comparable alternatives. Increasing alternate options as well as improvement in manufacturing and access is an area of high importance to improve RRMM outcomes.

## Introduction

Relapsed refractory multiple myeloma (RRMM) has a poor prognosis, with overall survival of around 6 months for penta-refractory patients, refractory to conventional therapy including immunomodulatory drugs (IMiDs), proteasome inhibitors (PIs), and CD38-directed therapy (1).

B cell maturation antigen (BCMA) is a novel treatment target for multiple myeloma due to its highly selective expression on plasma cells (2). The first-in-class BCMA chimeric antigen receptor T cell therapy (CAR-T) was idecabtagene vicleucel (ide-cel), approved in March 2021, for RRMM patients who progressed after ≥ 4 lines of therapy based on the results of the pivotal phase I/II KarMMa trial data. All patients had to be exposed to prior PIs, IMiDs, and CD38 targeting therapy as part of the FDA label. The overall response rate (ORR) was 73% and median duration of response (DOR) of 11 months in responders, and 20 mo in patients who had a stringent complete response (3, 4). Ide-cel manufacturing starts with leukapheresis, shipping of the T-cell apheresis product to the manufacturing facility, *in vitro* expansion, and transduction with a lentivirus vector (5). Purification and quality check is conducted prior to the release of the product and shipping back to the treating facility.

Despite the advancement in 2021 with ide-cel approval, the commercial manufacturing system has limited capacity, with limited production slot allocation nationally, and long manufacture times of at least 4 weeks (5–8). Long manufacturing and turnaround time increases the risk of mortality and morbidity in RRMM patients with rapidly progressive disease and potential deterioration before CAR-T infusion. In the KarMMa trial, 12 (8.5%) of the 140 patients who received leukapheresis were not able to receive the infusion of ide-cel. Only one of these was due to manufacturing failure, and the rest were secondary to patient condition and disease progression (4). The rollout of production slots for ide-cel has been relatively limited nationally.

In this report, we examine the outcomes of patients evaluated at our center for ide-cel between March to July 2021.

## Patients and methods

RRMM patients seen at the University of Kansas Cancer Center for ide-cel consultation between March 2021 to July 2021 were included. Slot availability and utilization from March to October 2021 were reported. Per institution policy, only those who met the KarMMa inclusion criteria were considered eligible for commercial CAR-T. Those that did not meet the inclusion criteria were not considered eligible. All patients considered eligible were refractory to the latest therapy. Factors such as patient fitness and comorbidities, availability of caregivers were taken into consideration. All eligible patients who were agreeable to CAR-T therapy were enrolled in the pharmaceutical company’s cell therapy portal. A “waitlist” was maintained with all eligible candidates. These patients were then presented at our Myeloma CAR-T planning weekly meeting, and the most appropriate candidates were selected for each ide-cel slot available. We evaluated the waitlist and compared the group that could not secure a CAR-T production slot (non-CAR-T group) and another group that secured a slot (CAR-T group). Patients who secured a slot but did not receive the ide-cel infusion were included in the CAR-T group. High-risk cytogenetics were per the International Myeloma Working Group (IMWG) criteria and included t(4;14), del (17/17p), t(14;16), t(14;20), and gain (1q) (9). Penta-refractory was defined as being refractory to two IMiDs, two PIs, and one CD38-directed therapy per IMWG criteria. The time to collection was defined as the time from consultation for CAR-T to leukapheresis. The time to manufacture was defined as the time from leukapheresis to CAR-T infusion. Lines of therapy at consultation was determined from diagnosis to initial consultation for CAR-T. Additional therapies for patients on the waitlist who could not secure a CAR-T production slot was not reported as line of therapy at consult. Bridging therapy was considered as therapy between leukapheresis to CAR-T infusion. Per institutional guidelines, bridging was held 2 weeks prior to cell infusion. Overall survival for both groups was calculated from the initial ide-cel consultation date. A retrospective chart review was performed to determine patient and disease characteristics, subsequent lines of therapy in the non-CAR-T group, and bridging therapy in the CAR-T group. The study was approved by the local institutional review board and conducted in accordance with the Declaration of Helsinki.

### Statistical analysis

Descriptive statistics were reported using medians for continuous variables. Comparisons of categorical variables were conducted using the Fisher’s Exact Test. A comparison of medians was conducted using a Mann-Whitney U test. Survival analysis was performed utilizing the Kaplan-Meier Survival estimator. All outcomes used an *a priori* two-sided p-value of 0.05 for significance. All statistical analyses were conducted in JMP^®^ (v15.1.0).

## Results

### Patient characteristics

**Figure 1:**
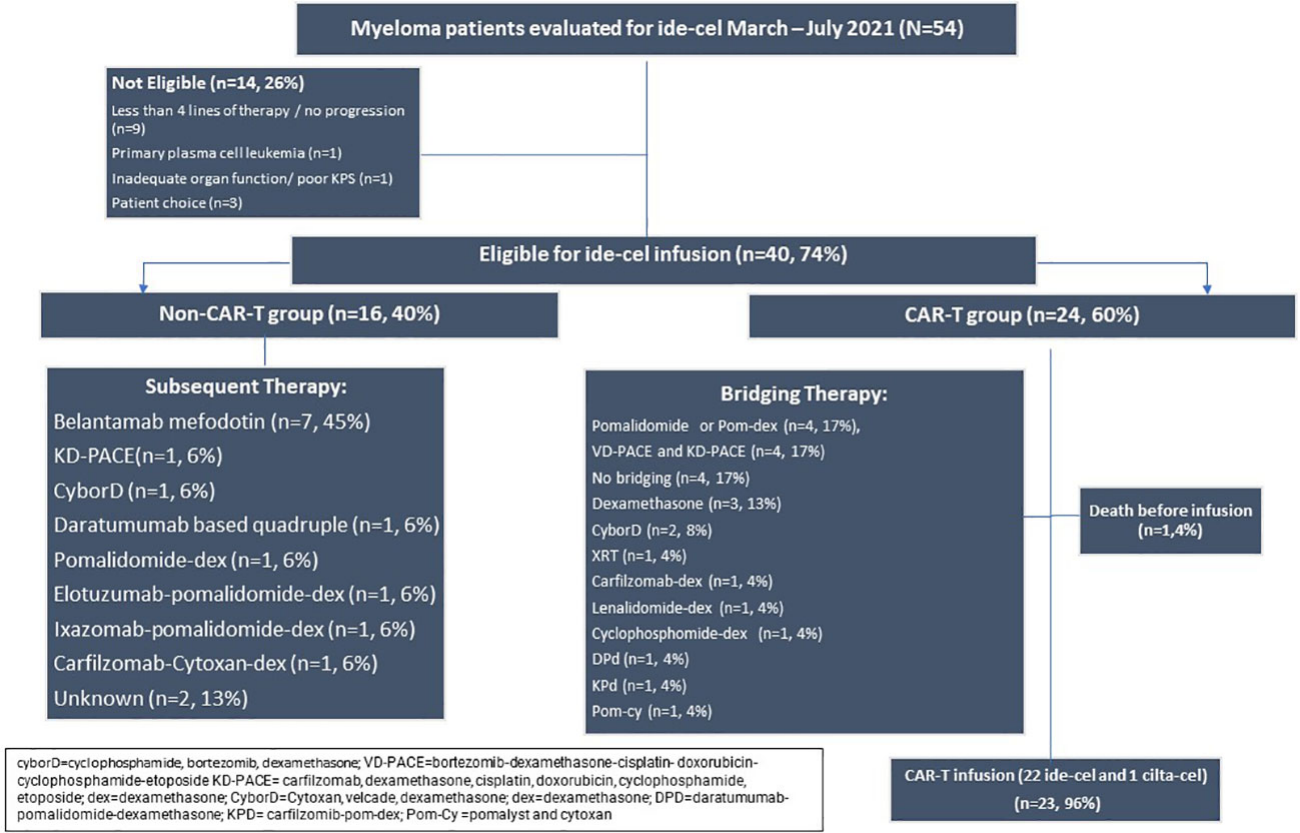
Consort of patients seen in consultation for standard of care ide-cel from March to July 2021.

Fifty-four patients were evaluated for ide-cel between March 2021 and July 2021. 14 patients (26%) were not eligible or chose not to pursue CAR-T therapy. There were 40 eligible patients who were included in further analysis. The median follow-up was 14 months (2-25mo). During this period, 16 (40%) were in the non-CAR-T group and could not secure a production slot while 24 (60%) patients secured a production slot (**Figure 1**). **Table 1** summarizes the demographics of these two groups. The median age was 61 (43-82) years. The groups were similar in time since diagnosis, penta-refractory status, extramedullary disease, high risk cytogenetics, and exposure to prior BCMA therapy (belantamab mafodotin). There was a median of 2 production slots per month from March-October 2021 (range 0-9). All slots were utilized.

**Table 1:** Patient and disease characteristics at consultation for CAR T.

Characteristics (n,%)	All patients (n=40)	Patients who Received Leukapheresis for BCMA CAR T (n=24)	Patients who could not receive leukapheresis for BCMA CAR T (n=16)	*p* value
Gender				
Male	26 (65)	16 (67)	10 (63)	1.000
Female	14 (35)	8 (33)	6 (38)	
Race				
Caucasian	28 (70)	19 (79)	9 (56)	0.126
African American	10 (25)	5 (21)	5 (31)	
Other	2 (5)	0 (0)	2 (13)	
Median Age at Consultation (Years, 25-75 Quartile)	61 (58-68)	61 (57-68)	61 (58-71)	0.709
Median Time from Diagnosis to Consult (Years, 25-75 Quartile)	5 (3-9)	6 (3-9)	4 (2-8)	0.192
Median Lines of Therapy at Consult (Number, 25-75 Quartile)	6 (5-8)	7 (5-8)	5 (5-7)	0.138
Myeloma Subtype				
IgG Kappa	12 (31)	8 (33)	4 (25)	0.925
IgG Lambda	8 (20)	5 (21)	3 (19)	
Light Chain (Lambda or Kappa)	7 (18)	4 (17)	3 (19)	
Other	13 (33)	7 (29)	6 (38)	
High-Risk Cytogenetics*	20 (50)	11 (46)	9 (56)	0.748
Extramedullary Disease**	12 (30)	7 (29)	5 (31)	1.000
Penta-refractory	32 (80)	21 (88)	11 (69)	0.229
Prior BCMA Therapy Exposure	10 (25)	7 (29)	3 (19)	0.711
Prior Autologous Transplant	30 (75)	20 (83)	10 (63)	0.159
Prior Allogeneic Transplant	3 (8)	3 (13)	0 (0)	0.255
Median Duration of Follow Up (Months, 25-75 Quartile)	14 (7-23)	22 (13-24)	8 (5-12)	**<0.001**
Median Time from Apheresis to Infusion (Days, 25-75 Quartile)	N/A	42 (37-49.5)	N/A	NC
6 Month Survival	31 (78)	22 (92)	9 (56)	**0.018**
Overall Survival (Months, 25-75 Quartile)	15 (9-NR)	NR (13-NR)	9 (5-12)	**<0.001**

*High risk Cytogenetics defined as t(4:14), t(14;16)t(14:20), gain 1q and 17p del. BCMA: B cell maturation antigen; **Defined as non-osseous extramedullary disease, BCMA=B cell maturation antigen.

### Non-CAR-T group

The alternate therapies in the sixteen patients who could not get CAR-T apheresis slots are listed in **Figure 1**. Belantamab mafodotin was the most frequent agent, used as monotherapy or in combination with dexamethasone (n=7, 45%).

### CAR-T group

All patients in this group received leukapheresis for ide-cel except one patient who received leukapheresis for ciltacabtagene autocel (cilta-cel). The median time from the consult visit to the collection for the CAR-T group was 38 days (8-703). The median time from collection to infusion was 42 days (34-132). There were 5 patients who secured a CAR-T production slot > 4 months from initial consultation. These patients received between 1-3 additional lines of therapy after initial consultation before securing a production slot. Bridging therapies are listed in **Figure 1**. The most commonly used bridging regimens included pomalidomide monotherapy or in combination with dexamethasone (n=4, 17%), bortezomib or carfilzomib with dexamethasone-cisplatin- doxorubicin-cyclophosphamide-etoposide (VD-PACE or KD-PACE) (n=4, 17%) and dexamethasone only (n=3, 13%). Four patients (17%) received no bridging therapy.

### Survival outcomes

**Figure 2:**
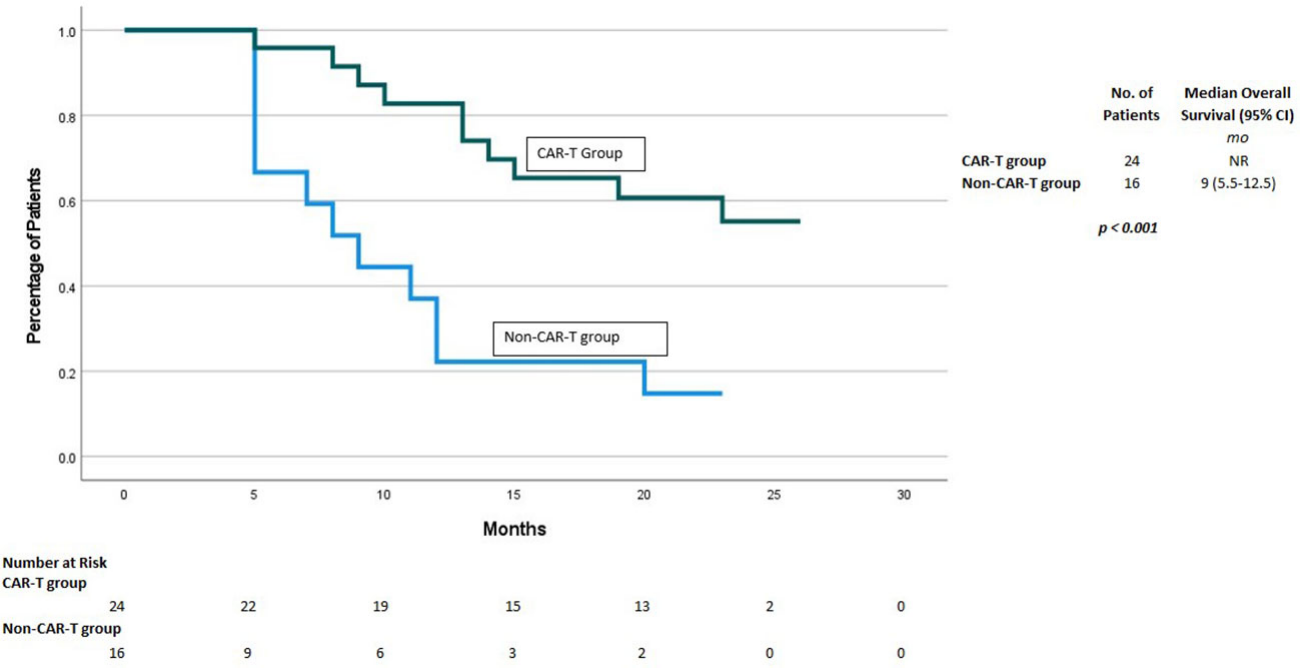
Overall Survival for CAR-T vs no CAR-T group.

The survival following CAR-T consult was lower among the non-CAR-T patients on the “waitlist” vs. the CAR-T group. With the median follow up of 14 mo, the mortality was 81% (13 patients) in the group that did not receive CAR-T, as depicted in **Figure 2**. Nine patients (38%) died in the ide-cel group due to progressive disease. One death occurred in a patient who was collected but did not receive an ide-cel infusion due to ongoing respiratory viral infection. The median OS was 9 mo in non-CAR-T group vs. NE in CAR-T group (p<0.001).

## Discussion

Ide-cel launched in March 2021 as the first-in-class BCMA Car-T cell therapy in RRMM, and remained the only BCMA-CAR-T till 2/2022 (3, 10). It is available at authorized treatment centers, with 73 centers across the United States offering this therapy as of July 2022 (11). One of the main challenges faced by many of the centers delivering ide-cel has been the allocation of only a few production slots per month due to manufacturing capacity limitations. The median slots per month for our institution was 2 slot (0-9) in 2021 which resulted in a long time to collection for those on the waitlist. We note a survival advantage seen in the patients on the waitlist who were able to receive ide-cel compared to standard alternate therapies in 2021. This is reflected in other studies as well with both ide-cel and cilta-cel (12–15).

An additional challenge for those who secure a slot is the long manufacturing time. The median vein-to-vein time in the CAR-T patients was 42 days (range 34-132 days). This represented time to manufacture except for two patients who were delayed due to infection. This is consistent with the known manufacture time in KarMMa trial of around 4 weeks from apheresis to infusion (4). One patient (5%) underwent leukapheresis but did not receive the infusion. Despite the long manufacture time, all other patients who underwent leukapheresis were able to receive the infusion. This appears to be better than the KarMMa data, where 12 (9%) patients did not receive the infusion after leukapheresis (4). Moreover, while manufacture failure was seen in 1% of patients in the KarMMa trial, we did not experience failure of manufacture in these patients in the real-world cohort (4). These observed differences observed may be secondary to limited sample size, differences in bridging strategies, and stringent patient selection adopted due to limited access.

The challenges with the production slot limitations and the long manufacture times has led to an ethical dilemma described by various institutions across the US (7). At our institution, we had stringent criteria for patient selection during the period evaluated. Only those patients who met the KarMMa inclusion criteria were considered. Even amongst these select patients, we had to choose the most appropriate candidates for the scarcely available slots. We had a weekly joint conference attended by the CAR-T and Plasma Cell Disorder teams at our institution to select appropriate patients, ideally who were fit, with a predictably slow pace of disease, with relatively low risk of morbidity and mortality during the manufacturing period. We took into consideration patient characteristics including performance status, cardiac function, creatinine clearance, comorbidities, and social support. We also had to take into consideration disease characteristics, such as lines of therapy, penta-refractory or triple-refractory status, number of transplants, prior BCMA therapy and clinical trial enrollment in last 24 months.

The RRMM field is dynamic and rapidly evolving with BCMA therapies. The approval of cilta-cel in February 2022 has increased the total slots per month, but opportunities for improvement exist (6). We describe in an editorial that the median number of production slots for BCMA CAR-T (including ide-cel and cilta-cel) increased at our institution in 2022 and 2023. In fact, in that report, we also now demonstrate that several production slots are not utilized, likely reflecting more readily available options with the approval of novel BCMA bispecifics such as teclistimab (16). Of note, belantamab is now withdrawn from the US market officially as of February 2023 (17). As BCMA CAR-T is being studied in earlier lines, and with various combinations, this novel class of therapy will likely be available for a broader patient population in the future (18, 19). Therefore, it is critical to prioritize research to improve manufacturing of ide-cel and other CAR-T products, and thereby increase utilization and access.

Several strategies are being proposed to counter the challenges in manufacturing and production seen with ide-cel as well as other autologous CAR-T products.

New CAR-T manufacturing platforms are being developed by numerous biotech companies that should allow for the scaling of manufacturing capacity while ensuring consistency in cell product properties (20, 21). These platforms too could be licensed to institutions once they receive full commercial approval to strengthen manufacturing. Decentralization and point of care manufacture at academic institutions is possible with at Good Manufacture Practice (GMP) – complaint closed automated systems which ensure reproducibility can improve supply chain and reduce manufacture time (22–24). Allogeneic BCMA CAR-T products are under investigation. These third-party products have the advantage of being readily available, “off-the-shelf”, with no manufacturing requirements. Several allogeneic BCMA CAR-T cell therapies are in development, including CTX120, CYAD-211 (25–27).

Moreover, non-CAR-T alternatives for BCMA therapy are now available, which will help improve outcomes for those who cannot secure a CAR-T slot. Belantamab mafodotin was the only available first-in-class immunoconjugate targeting BCMA, showing single-agent activity in the phase 1 DREAMM-1 study available during the period that we conducted the study (28). Belantamab mafodotin was the most favored alternative for patients who did not have prompt access to CAR-T in our study population, however, it was withdrawn from the US market In November 2022 (29). Teclistimab is the first BCMA directed bispecific antibody T cell engager to be approved in December 2022 (30). Other promising novel BCMA and non-BCMA directed bispecific antibody T cell engagers are in development and will broaden the non-CAR-T options for RRMM patients (31, 32).

Limitations of our study include that it was a retrospective review conducted at a single center with limited sample size. Many patients were referred from outside health systems and received bridging therapy and alternate therapies at different health systems. Although we captured survival data, we were not accurately able to capture granular details on number of cycles of therapy, and disease responses to the alternate therapies. Moreover, since patients with more aggressive disease biology were less likely to be selected for CAR-T given long wait times and manufacture times, we acknowledge a natural selection bias in survival differences.

We conclude that access limitations to ide-cel production slots existed in 2021 and there was a high mortality rate for patients who are on the waitlist and not able to secure a timely production slot. The survival differences may reflect the fact that patient selection was stringent given challenges with production slots and manufacturing times. The landscape of access to BCMA therapy has been dynamic over the years with the availability of cilta-cel and now with BCMA bispecific antibody T-cell engagers such as teclistimab. Prioritizing research to optimize manufacturing time is urgently needed to facilitate prompt access to BCMA CAR-T for RRMM patients.

## Data availability statement

The raw data supporting the conclusions of this article will be made available by the authors, without undue reservation.

## Author contributions

NA and LS conceived the study. SB and BG contributed to data extraction. WW was responsible for writing the protocol and performing data analysis. NA and WW interpreted the results and wrote the initial draft. All authors contributed towards the final manuscript and provided feedback on the report. All authors contributed to the article and approved the submitted version.
